# A Review of Microstrip Patch Antenna-Based Passive Sensors

**DOI:** 10.3390/s24196355

**Published:** 2024-09-30

**Authors:** Zain Ul Islam, Amine Bermak, Bo Wang

**Affiliations:** College of Science and Engineering, Hamad Bin Khalifa University, Doha 34110, Qatar; zais23380@hbku.edu.qa (Z.U.I.); abermak@hbku.edu.qa (A.B.)

**Keywords:** antenna, passive sensing, dielectric constant, permittivity, return loss, resonator sensors

## Abstract

This paper briefly overviews and discusses the existing techniques using antennas for passive sensing, starting from the antenna operating principle and antenna structural design to different antenna-based sensing mechanisms. The effects of different electrical properties of the material used to design an antenna, such as conductivity, loss tangent, and resistivity, are discussed to illustrate the fundamental sensing mechanisms. Furthermore, the key parameters, such as operating frequency and antenna impedance, along with the factors affecting the sensing performance, are discussed. Overall, passive sensing using an antenna is mainly achieved by altering the reflected wave characteristics in terms of center frequency, return loss, phase, and received/reflected signal strength. The advantages and drawbacks of each technique are also discussed briefly. Given the increasing relevance, millimeter-wave antenna sensors and resonator sensors are also discussed with their applications and recent advancements. This paper primarily focuses on microstrip-based radiating structures and insights for further sensing performance improvement using passive antennas, which are outlined in this study. In addition, suggestions are made for the current scientific and technical challenges, and future directions are discussed.

## 1. Introduction

The rapid advancement of sensing technology connects the physical world to the digital world, where sensors play a vital role [1]. These sensors can be widely categorized as either active or passive. The fundamental distinction between active and passive sensors lies in their energy sources and operational dependencies. The active sensors are powered by onboard batteries and are used for data acquisition and transmission over a longer distance. However, the power consumption of passive sensors is very low, which they acquire from the interrogated/incident signal [2].

**Figure 1:**
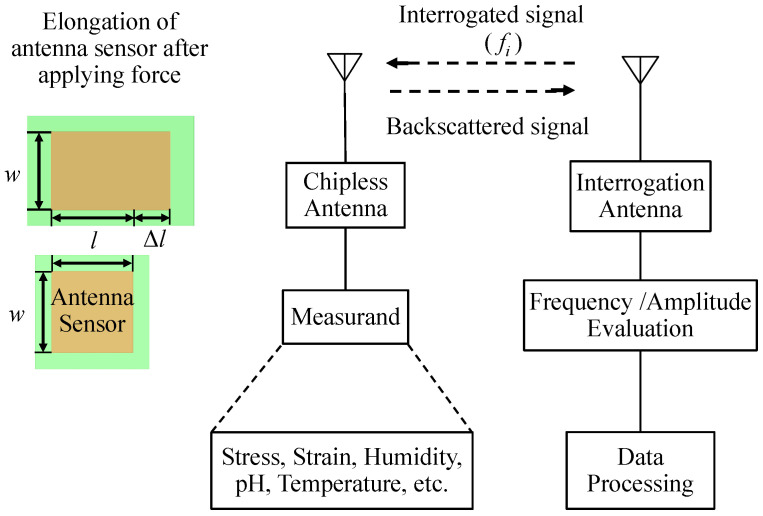
Illustration of an antenna-based sensing system.

Advanced manufacturing technology progressed fast over the past few decades [3,4], leading to the development of more compact sensors capable of sensing a broad range of physical, chemical, and biological targets [1]. Particularly, driven by powerful data analytics tools like machine learning, deep learning, etc., there is a rising demand for data captured by sensors to achieve a higher level of society intelligence, such as predictive maintenance, e-logistics, smart healthcare, etc. Because of the capability to perform wireless while purely passive (i.e., battery-free) sensing [2], the antenna-based sensor is becoming an interesting solution able to capture large quantities and high-quality data at a relatively low cost. When exposed to the target measurand, the sensing antenna’s material property (e.g., substrate material or material coated on the antenna surface) and dimension could change, which then alters the antenna’s radiation characteristics. After being interrogated with a fixed-frequency signal or a wide-band signal from a reader, as shown in Figure 1, the amplitude and frequency content of the reflected signal from the antenna can be analyzed to achieve the sensing function. Compared with other passive devices like surface acoustic wave (SAW) sensors [5], passive RFID [6], etc., antenna-based sensors offer several unique benefits like battery-less and chip-less operation, maintenance-free, low cost, fast deployment, and, mostly, disposable.

Hitherto, there are antenna-based sensors investigated in applications such as disease detection and remedies [7,8,9], wireless cough monitoring [10], non-invasive sweat sensing [11], blood glucose detection [12,13,14,15,16], salinity/sugar concentration sensing [17,18,19], and other chemical-related sensing [20,21,22,23,24]. They were also widely used to sense environmental parameters, including moisture/humidity [25,26,27], pH [28], temperature [29,30,31], ice accumulation [32], etc. Antenna-based sensors have also been applied for industrial sensing, such as corrosion detection [33], cement hydration sensing [34], and structural health monitoring [35]. Overall, efforts were made to apply antenna-based sensors in various applications, including healthcare, agricultural, environmental, and industrial sectors.

Although they exhibit appealing advantages, antenna-based sensors are limited by undesired ambient reflections and interference from other radiation sources in the scene [36,37]. A directive and narrow-band sensitive antenna can avoid some of these issues, while extra care must still be paid during deployment. In particular, the sensitivity of an antenna-based sensor highly depends on the substrate material selection and its physical geometry. Thus, it is crucial to understand the principles and trade-offs of designing high-performance antenna-based sensors. Hence, we aim to provide a technical review of the recent advancements in antenna-based sensor design. This paper revisits the main antenna design parameters affecting the sensing performance. In addition, different existing sensing mechanisms are reviewed, including frequency shift, return loss variations, and received signal strength, with their respective pros/cons discussed.

The rest of this paper is organized as follows. A historical overview of antenna-based sensors is described in Section 2. Section 3 revisits the working principles of how an antenna is used for sensing purposes and the parameters affecting the sensing performance. Different antenna-based sensing techniques are explained in Section 4. Section 5 explains the millimeter-wave (mmW) antenna sensors, resonator sensors, their applications, and recent advancements in this field. Design challenges and future work in this research area are discussed in Section 6, with a brief conclusion in Section 7.

## 2. Historical Overview of Antenna-Based Sensors

The use of antennas for sensing purposes is not new, as it was practiced after Heinrich Rudolph Hertz developed the first antenna in 1888. Initially, it was based on electric/magnetic field sensing to detect mines, metals, and weapons during the wars. Archaeological explorations and infrastructure analysis have also benefited from this technology. Radar-based sensing also uses antennas to transmit and receive electromagnetic (EM) waves. By analyzing the reflected wave/signal, radars can detect objects and determine their location, speed, and direction [38]. Specifically, ground penetrating radars employ antennas to sense the structures or objects buried in the ground [39].

Over time, antenna-based sensors have undergone significant technological advancements, finding applications in various fields. In 1995, a 1.5 GHz circular patch antenna was presented to detect the moisture content of sludge samples [40]. It was the earliest known attempt to use an antenna-based sensor for physical parameter sensing, i.e., moisture content. Depending on the antenna-based sensor’s effective dielectric constant changes, the sludge sample’s moisture content can be estimated. When the moisture level of the sludge sample was high, this approach could achieve a resolution of ∼0.3%. The realization of this notion was the beginning of the utilization of antennae for other environmental parameter sensing.

Later, antenna-based sensors designed together with radio frequency identification (RFID) technology were reported. In [41], one RFID tag was covered in a water-absorbing material, while the other was left open. The difference in turn-on power of these two RFID tags was used to calculate relative humidity. Later, a similar approach was utilized for displacement threshold sensing [42]. Temperature detection based on an antenna was also demonstrated using the temperature dependency of its dielectric material [43].

Recently, with the advancements in printed/flexible electronics, antenna-based sensors have been integrated into wearable devices to monitor health metrics [44]. Because of this, antenna-based sensors have transitioned from simple environmental detection to sophisticated biomedical applications [7,8,9], etc. For example, microwave imaging employing antennas can detect cancer tumors based on the dielectric constant contrast between healthy and malignant tissues [9]. Further innovations in this domain are anticipated as technology advances, making antenna-based sensors more efficient and versatile.

## 3. Antenna Basics and Design Considerations for Sensing Applications

**Figure 2:**
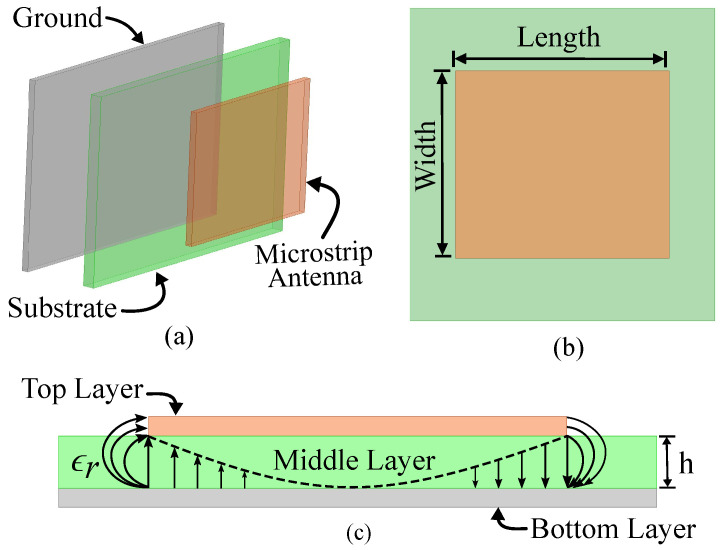
Microstrip patch antenna: (**a**) perspective view, (**b**) top view, and (**c**) side view [45].

The function of an antenna is to radiate and receive EM waves [45]. There are several types of antennas, such as wire antennae, parabolic reflector antennae, aperture antennae, waveguide antennae, etc. Among them, microstrip antennae are more popular for sensing applications as they are conformal [46], robust [47], small-in-size [48], inexpensive [31,49], and sensitive to its substrate dielectric variation. Therefore, we will briefly revisit the operation/property of a microstrip patch antenna shown in Figure 2 in the context of sensing applications. Particularly, the selection of proper antenna resonant frequency, impedance, and substrate material for different sensing requirements/conditions will be discussed.

A microstrip antenna is a thin and planar antenna consisting of a patch, typically rectangular or circular, placed on one side of a dielectric substrate with a ground plane on the other side [45]. The patch and ground are made of a conducting material, such as copper, while the substrate is typically a low-loss dielectric material. For a rectangular microstrip patch antenna shown in Figure 2, its resonant frequency $f_{r}$ is mainly determined by its width (*W*), length (*L*), and dielectric constant $\epsilon_{r}$ of the substrate, with the substrate height (*h*) also slightly affecting $f_{r}$ [45]. The simplified relationship between $f_{r}$ and different design parameters are [45]

(1) $$f_{r}=\frac{c}{W\sqrt{2(\epsilon_{r}+1)}}$$

and

(2) $$f_{r}=\frac{c}{2L\sqrt{\epsilon_{r}}},$$

where *c* is the speed of light. Therefore, even a small-size antenna-based sensor can reach gigahertz-level working frequency (e.g., 1 GHz, with 93 mm width, 73 mm length, and an $\epsilon_{r}$ of 4.25). With a pre-defined $f_{r}$, using a substrate with a larger dielectric constant allows a smaller sensor to be designed. Note, that other factors like feed position, fringing effects, and radiator shape also play important roles in antenna-based sensor design [45]. Selecting a proper $f_{r}$ is crucial during design, which involves the consideration of the application requirements, the dielectric properties of the object being sensed, and environmental conditions.

If the sensing target is located within a non-metal medium (such as soil [50], tissue [9], clothing [51], etc.), sufficient penetration of the interrogating and backscattered EM waves is required in that medium for effective sensing. EM wave penetration depth into a material is highly frequency-dependent because of the ionizing effect [52], with lower frequencies generally penetrating deeper. It is characterized by the skin depth (δ), representing the distance at which the wave’s amplitude decreases to about 36.8% of its original value [53], and is expressed as

(3) $$\delta =\sqrt{\frac{\rho }{\pi f_{i}\mu }}$$

where ρ is the resistivity of the medium (in Ohms/meter), $f_{i}$ is the interrogating wave frequency (typically the same as $f_{r}$), and μ is the medium’s magnetic permeability (in henry/meter). The skin depth increases for materials with high resistivity and low permeability, allowing for deeper penetration when required by the sensing application. Exceptionally, Terahertz (THz) waves can penetrate non-metal mediums [54] and allow fine-details detection, such as molecular level sensing with a nano-antenna [55].

If the sensor aims to detect fine details, such as displacement, higher interrogating and antenna resonant frequencies are often preferred. The Rayleigh criterion defines the best achievable spatial sensing resolution (Δx), which is inversely proportional to the incident wavelength ($\lambda_{i}$), with Δx ≈λ/2 [56]. It means an object can be properly detected when its size is larger or bigger than half of the interrogating wavelength. Higher frequencies also enable the use of smaller antenna sizes, resulting in improved spatial resolution with narrower beamwidth, higher gain, and improved directivity [57].

Besides the above considerations, the application environment could also affect the selection of $f_{r}$. Specifically, ambient temperature variation could cause substrate expansion/compression and alter $\epsilon_{r}$ [31]. A humid environment behaves like a lossy dielectric medium, attenuating the EM waves and altering the antenna radiation characteristics [58]. Dust particles in the air can introduce undesired signal attenuation and interferences [59]. Therefore, environmental factors that can potentially deteriorate the sensing performance must be considered when selecting the operating frequency $f_{r}$ and the sensor substrate material (discussed later).

The antenna impedance $Z_{a}$ is another important design parameter that should also be properly selected during the sensor design. It is a complex quantity represented as $Z_{a}$ = $R_{a}$ + *j$X_{a}$*, where $R_{a}$ is the resistive part and $X_{a}$ is the reactive part. Specifically, $R_{a}$ is the combined effect of antenna radiation resistance $R_{r}$ and loss resistance $R_{L}$ [45], which is typically frequency-independent. When the interrogated signal strikes the sensor, some of the captured power reradiates (backscatters) through $R_{r}$, and the remaining power dissipates/is lost as heat in the antenna’s conductive/dielectric elements through $R_{L}$ [45]. The imaginary part $X_{a}$ consists of capacitive and inductive reactances, which are frequency-dependent, and contribute to the stored energy in the near field region of the antenna [53].

For antenna-based passive sensors, a high impedance mismatch is desired to achieve stronger backscattering, which is characterized by the reflection coefficient Γ [53]

(4) $$\Gamma =\frac{Z_{a}-Z_{o}}{Z_{a}+Z_{o}},$$

where $Z_{o}$ is the intrinsic impedance of free space. An antenna with high conductivity (i.e., low impedance with $Z_{a}≃0$) may achieve maximum reflection of the interrogated signal (e.g., |Γ|≈1 in the best case). Similarly, if $Z_{a}\gg Z_{o}$, maximum reflections/reradiation can also be achieved for passive sensing. In the case of $Z_{a}$ = $Z_{o}$, no reflection occurs due to perfect impedance matching. The reflections of an interrogated signal depend on the medium impedance for which $Z_{a}$ can be tuned/adjusted using the appropriate substrate material. Furthermore, the reflection of an interrogated signal is also highly dependent on the sensor surface (planar, curved, rough, smooth, etc.) and the angle of the incident wave [53].

Another parameter to describe the reflection behavior is return loss (RL, typically in decibels), the measurement of how much power ($P_{\mathrm{ref}}$) is being reflected to the transmitter (e.g., an interrogating wave power of $P_{\mathrm{in}}$) from the sensor antenna [60], described as

(5) $$\mathrm{RL}=10\log\left(\frac{P_{\mathrm{in}}}{P_{\mathrm{ref}}}\right).$$

Low return loss values indicate high reflected power levels, leading to stronger signal reception at the reader side for better passive sensing performance.

Antenna impedance is generally a function of frequency, but a few of the antenna parameters and other factors also influence it. For example, the antenna’s geometry, physical dimensions, excitation method, and feed point directly alter the impedance [61]. Such as, for a rectangular/square microstrip patch sensor, the current is low near the ends and gradually increases while moving toward the center resulting in low impedance around center [45]. EM coupling from other radiation sources alters the surface current distribution and introduces additional capacitance or inductance, which noticeably changes antenna impedance [62]. The presence of nearby objects in the proximity of an antenna highly influences the antenna impedance and radiation characteristics [63,64,65]. In general, for better signal reception and reradiation, antenna impedance plays a vital role which should be taken into account for an efficient sensor design.

The selection of a substrate material also plays an important role in the antenna radiation characteristics. The major substrate physical properties that affect the antenna’s efficiency and sensitivity are conductivity, loss agent, etc., as discussed below.

- Conductivity σ

The conductivity of a material directly affects the antenna’s sensitivity. It is related to the incident electric field (**E**), which produces the antenna’s surface currents, by the following formula [53]

(6) $$\sigma =\frac{J}{E}=\frac{1}{\rho }$$

where **J** is the current density and ρ is the resistivity of a substrate material. For a low-resistive material, a minor incident **E**-field produces good surface currents and vice versa. A significant amount of current produced by the small **E**-field, resulting in the generation of a backscattered signal, corresponds to a more sensitive antenna. In general, higher conductivity materials exhibit lower ohmic losses (R_*o*_) and can efficiently capture and transmit EM signals, which results in an increased sensitivity performance.

- Loss tangent (tanδ)

The loss tangent is a key parameter that quantifies the antenna-based sensor’s substrate dielectric losses and provides information about how much energy is absorbed by the dielectric material during signal reception and reradiation. It is a measure of received energy loss, commonly as heat, in a dielectric material due to factors like dielectric losses and conduction losses affecting the overall antenna efficiency. It can be defined as the ratio, tan(δ) = *ϵ*″/*ϵ*′, of the imaginary part of the complex permittivity (ϵ″) to its real part (ϵ′) [66]. The ϵ′ represents how much energy from the **E**-field component of an interrogated EM signal is stored in a material while the ϵ″ is a measure of how dissipative or lossy a material is to that incident **E**-field [67].

To improve the antenna’s efficiency, high-quality substrate material with low-loss tangent values should be used. Materials with lower loss tangents exhibit lower energy dissipation and, therefore, can yield better radiation efficiency and backscattering. The loss tangent values of different materials with other electrical, mechanical, and chemical properties are provided in the specific data sheet of that particular material/substrate. Loss tangent values of some common materials [68] are presented in Table 1. Materials with low values of loss tangent, such as Rogers laminates, perform well at higher frequencies than high loss tangent value materials, for example, FR402.

The appropriate substrate material selection is the first and most important consideration for different sensing scenarios. For instance, flexible substrates with good mechanical stability of bending and stretching, such as textile [69], paper [70], and plastics or polymer substrates [71], are best suited for sensing scenarios where antenna mounting is desired on curved or bent surfaces. Due to high thermal stability, different ceramic-based substrates, such as Hexagonal Boron Nitride (hBN) and alumina ceramic (AL_2_O_3_), are the best choice for high-temperature sensing [72]. For implantable biosensing, biocompatible substrate materials, such as zirconia, Silastic MDX-4210 Elastomer, Alumina, Titanium Nitrite (TiN), etc., are the opposite option as they do not react adversely with the surrounding tissues [73].

## 4. Existing Antenna-Based Sensing Mechanisms

**Figure 3:**
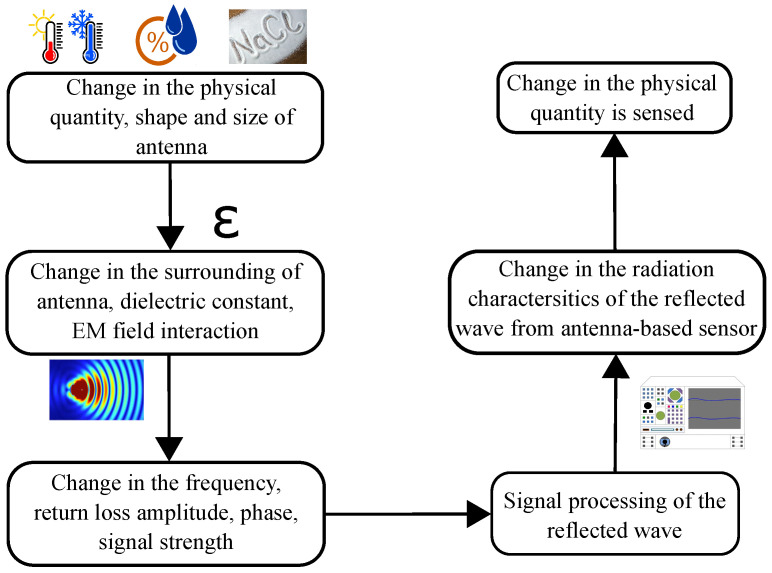
Illustration of antenna-based sensing mechanism.

For an antenna-based sensor, changes in the measurand can alter its radiation characteristics because of the antenna shape/dimensions and/or dielectric property variations caused by the target measurand, as illustrated in Figure 3. Typically, the measurand could affect the antenna radiation characteristics from several perspectives, while most often, only one or two changes are more significant than the rest. Therefore, four indicators are popularly employed to achieve such a sensing function, including

- sensing based on resonant frequency shift,
- sensing based on return loss variation,
- sensing based on phase variation,
- sensing based on both resonant frequency shift and return loss variation,
- and sensing based on received signal strength.

These sensing mechanisms are illustrated in detail in the following, with their technical tradeoffs discussed regarding the applicability and requirement of the reader.

### 4.1. Sensing Based on Frequency Shift

**Figure 4:**
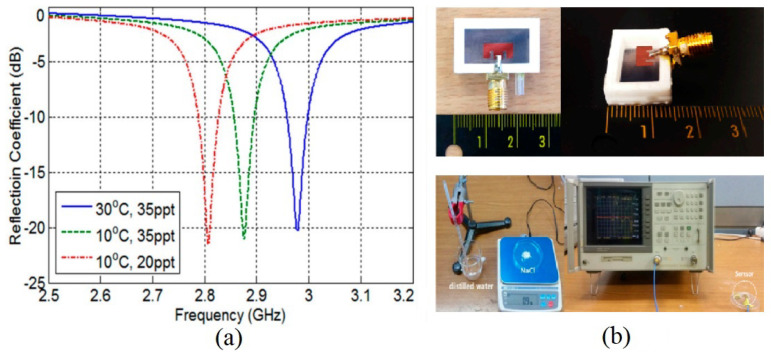
(**a**) Sensing based on frequency shift with respect to salinity level and temperature, (**b**) fabricated prototype and experimental set up [17].

Operational frequency is one of the primary parameters of an antenna-based sensor that performs sensing functionality, and it depends on the antenna’s physical size, shape, structure, material/substrate, etc. The length/width of an antenna-based sensor, shown in Figure 2, is the main parameter that directly controls the operational frequency. Equations (1) and (2) show as the length, width, or dielectric constant of the substrate increases, the center frequency ($f_{c}$) shifts towards lower frequency and vice versa. By utilizing this phenomenon, when a target quantity alters the antenna-based sensor’s parameter related to the resonant frequency, the interrogator module senses the change in target quantity based on frequency shift. In Figure 4 [17], a shift in the resonant frequency with respect to salinity level and temperature is shown, which is a perfect example of sensing based on frequency shift [17].

Although the frequency shifts with the directly related parameters mentioned above, it is also affected by ambient interference, which may cause errors in the reading/sensing at the interrogator side. Another performance degrading factor associated with the frequency shift sensing mechanism is that the reader must scan the whole wideband spectrum or at least around the original $f_{r}$ at high resolution. As a result, the overall sensing may experience some delay during frequency scanning. However, the utilization of this technique with change in the dielectric constant is investigated for various applications. For example, in [9] an implantable antenna-based sensor is developed, to operate at 2.45 GHz, for the diagnosis of the occurrence and recurrence of cancer tumor cells. The sensing principle of the proposed s-shaped monopole antenna is to monitor the pathological changes of the human breast tissues and the frequency response of the antenna. When the surrounding tissues permittivity changes due to cancer cell development, it directly affects the antenna’s resonant frequency. As a result, the human breast tissue is characterized by monitoring the resonant frequency shift/change.

Another useful application of antenna-based sensors is proposed in [31], for the passive temperature sensing. The proposed antenna utilizes the phenomenon of shift/change in the resonant frequency with the temperature variation. Two major factors affect the resonant frequency of antenna sensors. One is the change in the substrate dielectric constant, and the second is the metal thermal expansion with temperature variation. The sensing range of the proposed antenna sensor is from −40 °C to 125 °C with a reported sensitivity of 347.45 KHz/°C [31]. Similar to the above examples, sensing based on frequency shift with the change in the physical dimensions of an antenna-based sensor has also been used for pressure sensing [74], strain evaluation [35], etc.

### 4.2. Sensing Based on Return Loss Variation

**Figure 5:**
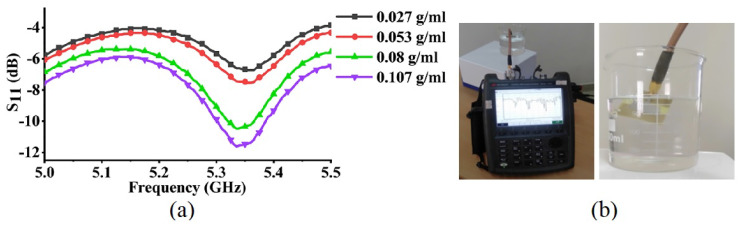
(**a**) Measured sugar concentration sensing based on Return Loss variation, (**b**) experimental setup [19].

An antenna-based sensor’s radiation characteristics are analyzed in terms of return loss amplitude, shown in Figure 5 [19]. Return loss up/down movement and amplitude variation can be utilized as a sensing parameter [19], which is related to impedance match/mismatch between the antenna-based sensor, its surroundings, and interrogated signal reflections based on measurand changes. From Figure 5, typically the reflection and transmission of an interrogated signal are analyzed with respect to −10 dB return loss value [75]. As the return loss value decreases below −10 dB the reflections lessen and transmission quality improves (undesirable). However, higher return loss values near and above −10 dB indicate higher impedance mismatch and stronger signal reflections, which are desirable for antenna-based passive sensing. Considering these return loss values, the antenna’s operating bandwidth is determined as below −10 dB.

(7) $$Bandwidth=f_{u_{-}\left(-10dB\right)}-f_{l_{-}\left(-10dB\right)}$$

where $f_{u_{-}\left(-10dB\right)}$ is the upper frequency, and $f_{l_{-}\left(-10dB\right)}$ is the lower frequency. When compared to the bandwidth’s center frequency ($f_{c}$), an antenna’s quality (Q) factor indicates how wide its bandwidth is. The quality factor of a sensing antenna is calculated by the following formula [45]

(8) $$\mathrm{Quality}\;\mathrm{Factor}\left(QF\right)=\frac{f_{c}}{f_{u}-f_{l}}.$$

A higher Q-factor antenna will have a lower susceptibility to interference, which means it can maintain its signal-to-noise ratio better in the presence of noise. This is because it has a narrower bandwidth and higher selectivity, which allows it to filter out unwanted signals more effectively.

Sensing with return loss variation has the advantage of scanning just a single frequency band, which makes the overall sensing faster than the sensing with frequency shift. Although the overall system performance also depends on the reader antenna gain and radiation power, it does not affect antenna sensor information reading as these influences are ground truth. Utilization of return loss variation based on passive sensing is investigated in various fields of interest, presented in Table 2, such as a chemical concentration in a liquid, etc.

An antenna can work as a sensor for analyzing water quality and the concentration of sugar (C_12_H_22_O_11_) and salt (NaCL) in it by measuring the changes in the electromagnetic properties of the water and the variation of return loss. Naturally found water has many impurities in it in the form of different particles, salt, sugar, etc. The presence of dissolved substances/impurities in the water causes changes in the dielectric properties of water, which in turn, affects the amount of energy reflected back by the antenna. There is a proportional relationship between the return loss and conductivity of water; as conductivity increases, return loss increases, too. The number of free water molecules and the conductivity parameter affect the water’s characteristics. In the case of salt, as the salinity increases, the conductivity of water increases, resulting in a higher return loss value of the antenna sensor. However, the behavior is the opposite in the sugar-dissolved solution. In sugar-mixed water, there is a deficiency of ions in water so water conductivity goes down, resulting in a decrease in return loss correspondingly. Similarly, the quality analysis of different water samples is conducted based on the return loss of an antenna sensor corresponding to a change in conductivity of the water sample and dissolved ion concentration [19].

### 4.3. Sensing Based on Phase Variation

**Figure 6:**
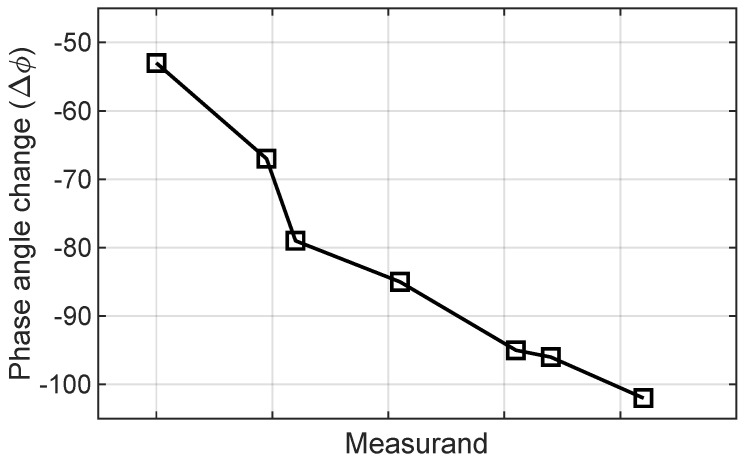
Sensing based on phase variation [77].

Sensing using an antenna phase angle variation is based on the principle that changes in the environment, surrounding medium, or the object/measurand being sensed can alter the phase of the reflected/received signal compared to the transmitted signal. Generally, the received signal (S) is represented in a complex-valued form as S = A_*r*_(t) · $e^{j\phi_{r}\left(t\right)}$, where A_*r*_(t) represents magnitude (the amplitude of the received signal) and $\phi_{r}\left(t\right)$ represents the phase angle of the received signal. The $\phi_{r}\left(t\right)$ is the relative position of the EM wave in its oscillatory cycle at any time (t) which varies due to changes in the environment, measurand, or propagation medium. However, a general relationship can be established as $\phi_{r}\left(m\right)$ = f(m), where f(m) is a function describing how phase angle changes in response to certain stimuli. Mathematically, this relationship could be linear, exponential, logarithmic, or any other functional form, as shown in Figure 6, depending on a specific application and the nature of the phase variation being observed. By analyzing the phase angle variations of the received signal, it is possible to extract information about the environment or the object being sensed over time.

Depending on the application, sensing can involve detecting the received signal’s phase change over time, frequency, or measurand quantity changes [76]. For example, in [77], metal crack detection is achieved by employing passive microstrip antenna sensors. It utilizes the change in the input impedance and reflected signal phase for analyzing the crack location and dimensions (length, width and depth). The phase variation-based sensing is relatively more advantageous as it eliminates the need for wide-range frequency scanning, and also does not get affected by the varying power levels as compared to sensing based on RL variations.

Another useful application of sensing based on phase variation is proposed in [78] for passive temperature sensing. The proposed design is a passive integrated surface acoustic wave (SAW) antenna sensor. Upon reflection by multiple reflective gratings on the SAW device, a set of reflected electromagnetic signals with varying delay times are produced. The temperature information could be extracted by comparing the phase shift of adjacent echoed signals produced by SAW reflective grating. The impact of temperature fluctuations on the resonance frequency could be ascertained. Similar to the above example, sensing based on phase shift is also employed for monitoring the change in the physical height of dielectric substrate and characterization [79].

### 4.4. Sensing Based on Both Frequency Shift and Return Loss Variation

**Figure 7:**
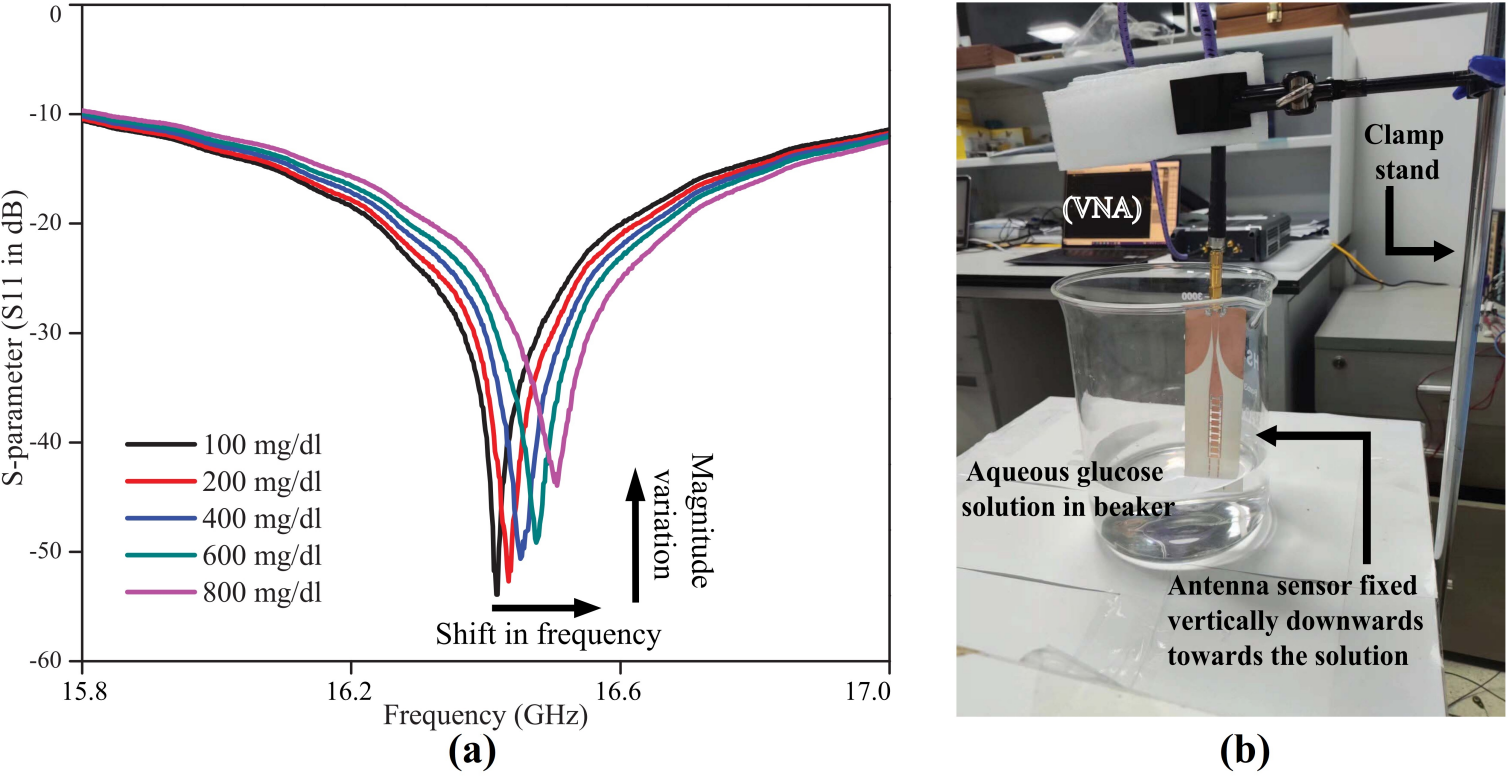
(**a**) Measured frequency shift and return loss variation response for glucose concentration sensing (**b**) experimental setup [14].

Some measurand quantities may cause an antenna-based sensor’s frequency shift or return loss variations separately. Practically, in most applications, both techniques work together for sensing purposes, as shown in Figure 7. This is because whenever an antenna’s dimensions/structure or dielectric properties are changed, it ultimately brings changes in frequency and return loss simultaneously. For example, when an antenna undergoes some bending/crumpling, it may change both frequency and return loss depending on the bending plane, angle, and direction. The effects of bending or curvature of different substrates have a different impact on radiation characteristics at different bending angles [80]. Material characteristics and dielectric constant are also important as different materials such as PVC, PET, and Teflon exhibit different changes in the frequency and return loss at the same frequency and bending angle [81].

The dielectric constant of the substrate material affects the velocity of electromagnetic waves within the material. When the antenna substrate is bent or deformed, it can change the effective dielectric constant experienced by the electromagnetic waves, thereby affecting the propagation speed. This alteration in the dielectric constant can lead to a change in the antenna’s resonant frequency and impedance matching. Bending the antenna substrate may affect the electromagnetic coupling between different parts of the antenna structure or between the antenna and nearby objects. This can lead to changes in the impedance and radiation characteristics of the antenna, causing variations in its resonant frequency as well as return loss. With only one measurand quantity, the antenna’s several parameters may change, resulting in a change in signal quality and resonant frequency.

Sensing with both frequency shift and return loss takes more time for frequency and amplitude scanning, which causes a delay in overall sensing. However, it can detect any minor changes in the measurand that may bring any alterations in the radiation characteristics of the sensor. For example, in [35] a flexible planar dipole antenna is designed to operate at 9 GHz for structural health monitoring and strain sensing. Antenna deformation brought by mechanical strain alters the antenna’s impedance or dipole length, which changes the resonance of the backscattered electromagnetic signal. Due to impedance variations, the return loss changes occur, and an increase in the length of the dipole, due to applied strain, causes the frequency shift towards a lower frequency point. As a result, the mechanical strain applied on the wireless antenna sensor is characterized by simultaneously monitoring the change in resonant frequency and return loss.

Another useful application of an antenna-based sensor is proposed for monitoring the glucose concentration in an aqueous liquid solution [14]. The measurement clearly shows as the glucose quantity changes in the aqueous liquid, it produces a change both in frequency and return loss, as shown in Figure 7. In addition to glucose concentration monitoring for biomedical applications [82], sensing based on frequency shift and return loss variation has been employed in literature for various other applications, including non-invasive sweat-sensing [11], moisture sensing in grains [26], etc.

### 4.5. Sensing Based on Received Signal Strength

Received signal strength indicator (RSSI) is a metric used to examine the power levels of a reflected signal from the antenna-based sensor that the interrogator antenna receives. It provides insights into the distance (*R*) between the antenna-based sensor and reader module, the level of interference from other radiation sources, and medium losses [83]. Wireless passive sensing based on RSSI involves leveraging the changes in the received signal spectrum characteristics caused by the measurand quantity [84].

A common empirical model for estimating RSSI is the Friis transmission equation, which provides a basic relationship between input (transmitted) power, distance, and received signal strength. RSSI-based signal quality is generally measured in dBm, which can be deduced as follows:

(9) $$P_{r}=\frac{P_{t}\cdot A_{e}}{4\pi R^{2}}$$

where $A_{e}$ is the effective aperture area of the receiving antenna which can be represented in terms of the physical dimension (*D*) of the antenna and wavelength (λ) as follows,

(10) $$A_{e}=\frac{\pi D^{2}}{4}=\frac{\pi {\left(\lambda /2\right)}^{2}}{4}=\frac{\pi \lambda^{2}}{16}.$$

Now, substitute the expression for $A_{e}$ back in Equation (9) which becomes

(11) $$P_{r}=\frac{P_{t}\left(\pi \lambda^{2}/16\right)}{4\pi R^{2}}=\frac{P_{t}\lambda^{2}}{64R^{2}}=\frac{P_{t}c^{2}}{64R^{2}f^{2}}.$$

To express $P_{r}$ in dBm (RSSI), take the logarithm on both sides and multiply by 10 as follows,

(12) $$10\log_{10}\left(P_{r}\right)=RSSI\left(dBm\right)=10\log_{10}\left(\frac{P_{t}c^{2}}{64R^{2}f^{2}}\right)$$

(13) $$RSSI\left(dBm\right)=10\log_{10}\left(P_{t}\right)+10\log_{10}\left(\frac{c^{2}}{64R^{2}f^{2}}\right)$$

As *c*^2^/64 is a constant, the above equation could be simplified further:

(14) $$RSSI\left(dBm\right)=P_{t}\left(dBm\right)-20\log_{10}\left(R\right)-20\log_{10}\left(f\right)+20\log_{10}\left(\frac{c^{2}}{64}\right)$$

The above equations provide a simplified model and assume idealized conditions without interference. Therefore, using the empirical model of the Friis equation may require calibration and adjustments based on actual measurements in the specific environment where experiments are conducted.

**Figure 8:**
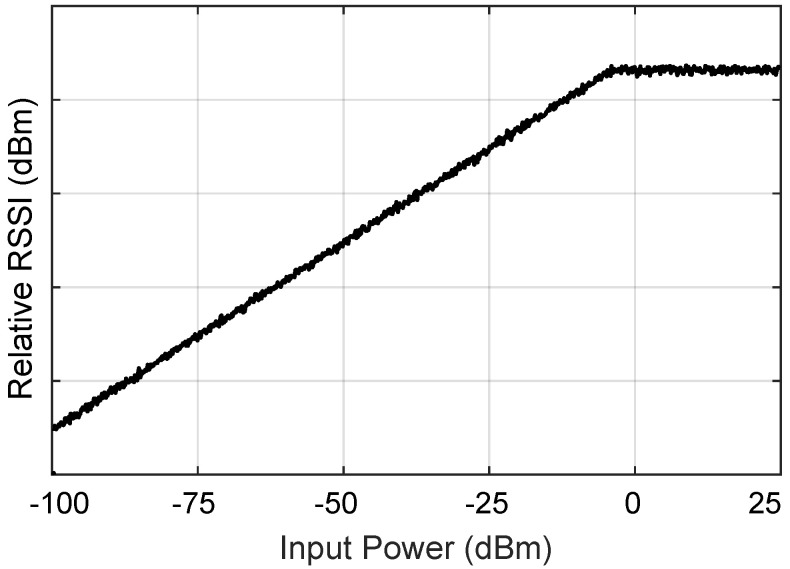
RSSI-based sensing mechanism [86].

It can be noticed that RSSI decreases with increased distance, and the received signal strengthens with increasing input power (14). However, after a certain point of input power, the RSSI level is constant even with more increasing input power, as shown in Figure 8. It may happen because receivers can handle a maximum power level without distortion. When the input power exceeds this level, the receiver’s components can saturate, causing the RSSI reading to remain constant or even decrease. Many systems have automatic voltage control circuits that adjust the voltage to maintain a constant received power level [85]. So, it is essential to understand the specific system’s architecture and components to pinpoint the exact cause of a constant RSSI beyond a certain input power level.

**Figure 9:**
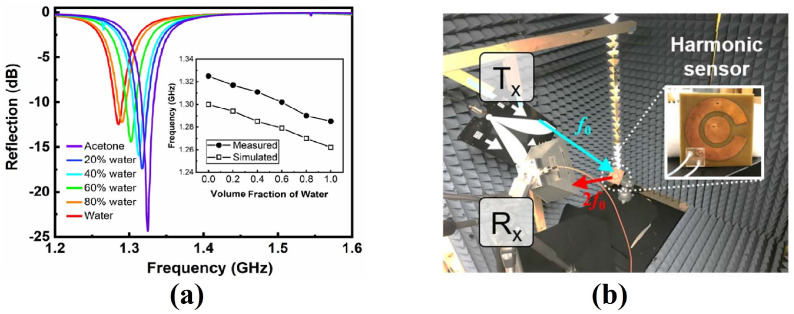
RSSI-based sensing mechanism (**a**) measured frequency response (**b**) experimental setup [23].

The utilization of RSSI-based sensing has been investigated in various applications. For example, a printed dipole antenna, with graphitic coating, is proposed for humidity level detection using an RSSI-based scheme [10]. The proposed antenna is tested under controlled conditions inside a gas chamber. When the water molecules are exposed to the antenna, the impedance of the coating changes, resulting in the impedance change in the antenna. As a result, the variations occur in the reflected signal power and indicate the humidity change at the interrogator side. Another useful application utilizing the RSSI-based sensing is proposed in [23] for liquid density monitoring. As shown in Figure 9, the proposed harmonic sensor experiences a shift in the frequency of reflected waves corresponding to a liquid mixture of water and acetone.

The antenna-based sensing is mainly performed by observing the change in the frequency component of EM signal in terms of frequency shift, amplitude alteration, or phase change. The required signal processing generally depends on the field of application. However, signal processing steps for antenna-based sensing generally include proper signal reception, data acquisition, and applying a filter to remove noise from the signal. Then, scattering parameter measurement or signal strength measurement is performed over a range of frequencies around the transmitted signal frequency. These measurements of the received signal are compared with the reference signal and based on the change in frequency, amplitude, phase and/or signal strength the sensing is performed. The final step involves relating the change in measured frequency response to the specific change in the measurand being sensed. The approaches of frequency shifting, return loss variation, and RSSI described above can be used separately or together for sensing purposes. Table 2 provides a variety of antennas using these sensing techniques. Different frequency bands are used for it, such as single band, wide band, ultra-wideband, extended ultra-wideband, etc.

**Table 2:** List of antenna sensor.

Ref.	Sensing Technique	Antenna Type	Operating Frequency	Substrate	Size	Target Application
[7]	Frequency shift	Microstrip Array	1.108 GHz	RO4003C	125 × 51 × 1.52 mm^3^	Cancer tumor
[17]	Frequency shift	Microstrip Patch	2.9 GHz	Silicon	20 × 10 × 5 mm^3^	Salinity sensing
[9]	Frequency shift	Microstrip antenna	2.45 GHz	RT6010	13.2 mm^3^	Cancer tumor
[31]	Frequency shift	Microstrip Patch	2.42 GHz	FR-4	56.2 × 70 × 1.6 mm^3^	Temperature sensing
[87]	Frequency shift	DRA	5.25 GHz	FR-4	30 × 30 × 1.6 mm^3^ r = 6.35 mm, h = 9 mm	Liquid chemical detection
[88]	Frequency shift	DRA	4.725 GHz	Rogers RT/Duroid 5880	60 × 60 × 1.52 mm^3^, r = 10 mm, h = 10 mm	Glucose sensing
[89]	Frequency shift	DRA	4.32–4.5 THz	silver-plated Arlon AD410	62 × 62 × 2 μm^3^, r = 28 μm, h = 10 μm	Hemoglobin sensing
[20]	RL variation	Microstrip Patch	3.6 GHz	FR-4	60 × 65 × 1.6 mm^3^	Methanol detection
[22]	RL variation	Microstrip Patch	1–6 GHz	Rogers TMM10i	N/A	Chemical vapor detection
[32]	RL variation	Microstrip Patch	3.927 GHz	FR-4	20 × 22.5 mm^2^	Ice thickness
[19]	RL variation	Microstrip Patch	3.2–10.6 GHz	FR-4	33.7 × 27.5 × 0.8 mm^3^	Salinity/sugar sensing and water quality
[77]	Phase variation	Microstrip antenna	2.83 GHz	Rogers 3010	27 × 25 mm^2^	Metal crack detection
[79]	Phase variation	Microstrip antenna	2.45 GHz	Rogers RO3010	15.5 × 6.25 mm^2^	Metal crack detection
[76]	Phase variation	Microstrip antenna	2.45 GHz	Rogers RO3010	15.5 × 7.6 mm^2^ 15.5 × 6.25 mm^2^	Metal crack detection
[11]	Frequency shift + RL variation	Microstrip Patch	2–4 GHz	Cellulose filter paper	60 × 50 mm^2^	Non-invasive sweat sensing
[14]	Frequency shift + RL variation	Microstrip Patch	10–25 GHz	Rogers RO 4003C	150 × 42 × 0.5 mm^3^	Aqueous glucose sensing
[26]	Frequency shift + RL variation	FSS Antenna	1–6 GHz	PF-4	60 × 28 × 50 mm^3^	Moisture content in grains
[35]	Frequency shift + RL variation	Microstrip Dipole	9 GHz	Polymer	17.2 × 15.6 mm^2^	Mechanical stress and strain
[10]	RSSI	Microstrip antenna	1.5/3 GHz	Roger 5880	65 × 35 × 0.5 mm^3^	Cough monitoring
[27]	RSSI	Microstrip Dipole	2.4–2.48 GHz	Paper/Silver ink	10 × 48 mm^2^	Relative Humidity (RH)
[23]	RSSI + Frequency shift	Microstrip Patch	1.3 GHz and 2.6 GHz	FR-4	85 × 85 × 1.6 mm^3^	Liquid density sensing
[21]	RSSI + Frequency shift	Microstrip Monopole	1.2/1.3 GHz and 2–6 GHz	Rogers 5880	100 × 70 × 0.5 mm^3^	Aqueous solution monitoring
[24]	RSSI + + Frequency shift	Microstrip Patch	2.86 GHz and 5.72 GHz	FR-4	25 × 12 × 1.5 mm^3^	Water level sensing in a liquid (aceton)

## 5. Antenna-Based Sensors and Resonator Sensors

Antenna-based sensors are not limited to microstrip antenna sensors for microwave sensing; rather, they have a wide range of applications in millimeter-wave (mmWave) antenna sensors and resonator sensors. In this section, mmWave antenna-based sensors and resonator sensors are discussed with a few of the applications reported in the literature.

### 5.1. mmWave Antenna-Based Sensors

The mmWave antenna-based sensors, operating within the frequency range of 30 GHz to 300 GHz [90], have emerged as pivotal tools in various high-precision sensing applications due to their propagation characteristics, which are crucial for detailed imaging and accurate measurements in diverse technical fields. These sensors exploit the properties of mmWave frequencies, such as short wavelength and high frequency, to achieve high spatial resolution and penetration capabilities, making them suitable for a wide array of applications.

One prominent application of mmW antenna sensors is in automotive radar systems [91,92]. These sensors are integral to advanced driver-assistance systems and autonomous vehicles, where they provide critical functionalities such as collision avoidance, adaptive cruise control, and lane-keeping assistance [93]. The high resolution of mmWave radar allows for precise detection of objects, their relative speed, and distance [94]. This reliability is paramount for ensuring safety and enhancing the operational efficacy of autonomous driving technologies.

Furthermore, mmWave sensors are extensively used in security and surveillance systems. Their ability to detect and image concealed objects makes them invaluable in airport security, where they are used in body scanners to identify potential threats without physical contact [95]. The high penetration capability and fine resolution of mmWave sensors enable the detection of non-metallic objects, contributing to enhanced security measures and streamlined screening processes [96].

Millimeter-wave antenna-based sensors are revolutionizing various sectors by providing high-resolution, reliable, and versatile sensing capabilities [97]. Their applications in automotive radar, industrial automation, security, telecommunications, and medical imaging highlight their critical role in advancing technology and enhancing operational efficiencies across multiple domains [98].

Recent advancements in millimeter-wave (mmWave) antenna-based sensors have been driven by innovations in signal processing and integration technologies. One of the notable advancements is the integration of mmWave sensors with silicon-based technologies, such as Complementary Metal-Oxide-Semiconductor (CMOS). These integrations have enabled the production of highly compact, low-cost, and energy-efficient mmWave sensors [99]. Silicon-based mmWave sensors have shown exceptional promise in high-frequency performance while maintaining manufacturability at scale, which is crucial for consumer electronics and automotive industries [100]. More progress has also been seen with the significant improvements in signal processing algorithms and techniques used for enhancing the data interpretation capabilities of mmWave sensors. Furthermore, machine learning and artificial intelligence (AI) techniques have been integrated into mmWave sensing systems to improve object detection, classification, and tracking [101].

### 5.2. Resonator Sensors

Resonator sensors are a type of sensor that operates based on the principle of resonance, where they detect changes in a physical quantity by measuring the shifts in the resonance frequency or resonance characteristics of a sensing system. These sensors exploit the interaction between the resonator structure and the surrounding environment, leading to a change/shift in the resonant frequency. Recent developments in metamaterial structures, such as split ring resonators (SRR) and microstrip resonator sensors, have focused on improving sensitivity, selectivity, and integration for a wide range of sensing and communication applications [102,103,104].

Microstrip-based resonator sensors leverage the properties of microstrip technology, which involves conducting strips on a dielectric substrate to detect changes in various physical quantities based on the complex permittivity extraction. The most common microstrip-based resonators reported in the literature are microstrip ring resonators [105], split ring resonators (SRR) [106], complementary split ring resonators (CSRR) [107], etc. The utilization of these resonators with changes in dielectric constant and frequency alteration is investigated in various applications. For example, in [108] a dual sensing resonator based on two SRRs is designed to work at two different frequencies to realize high accuracy for liquid permittivity detection. By detecting a single tested sample using two frequency bands, this sensor reduces the measurement inaccuracy that may come with utilizing just one frequency. Furthermore, it has the ability to simultaneously conduct dual sensing on two liquid samples. The sensitivity for SRR1 and SRR2 is 16 MHz and 22 MHz, respectively. Also, the normalized sensitivities are 0.28 and 0.3 for SRR1 and SRR2, respectively. The ability of this newly created sensor to monitor the effects of environmental conditions, such as ambient temperature, by using one band as the reference and another band as the testing sample, is another benefit. However, these kinds of sensors face the problem of mutual coupling for which some more techniques, just as using a power divider, are employed.

Another useful application of CSRR-based dual-band sensor is proposed in [103], to operate at 2.45 GHz and 5.8 GHz, for the sensing of bi-mixture liquid concentration just like urea and milk or water with ethanol. A suitable capacitance is produced by the CSRR being etched onto the substrate’s ground, resulting in an induced magnetic field caused by inductance. The ground plane experiences a concentrated field distribution due to this combination of capacitance and inductance. For the testing of the liquid mixture, a pipette or tube is passed through a hole in the middle of the substrate and middle of the etched CSRR in which the liquid is poured. When the tube is filled, one frequency band exhibits the sensing functionality, and the other is used as a reference sample. Similar to the above examples, microstrip-based resonators are used in sensing applications for dielectric characterization [102], biochemical sensing [109], liquid sensing [110], etc. In Table 3, the normalized sensitivities of the reported resonator sensors are given with their respective sensing application.

## 6. Challenges and Future Work

Antenna-based passive sensing has emerged as a powerful tool for various industrial applications due to its inherent advantages like being battery-free, lightweight, and offering remote interrogation capabilities. However, there are still significant challenges that need to be addressed for wider adoption and further advancement.

Miniaturizing passive sensing antennas while maintaining or improving their performance presents a significant engineering challenge. Compact antennas are desirable for applications where space is limited or where portability is essential. For antenna miniaturization, there are size reduction techniques [115,116], but they come at the cost of design and fabrication complexities. As a result of trade-offs between antenna size and frequency, an antenna could only be reduced up to a certain limit in size so far. Additionally, conformability to non-planar surfaces is desirable for applications like pipeline monitoring or strain sensing on curved surfaces. Research on novel and miniaturized antenna designs utilizing flexible substrates and innovative fabrication techniques are needed [117].

Improving the sensitivity of antenna-based sensors, particularly for weak signal detection, is crucial for applications like early-stage crack detection or monitoring subtle environmental changes. Strategies like incorporating novel materials [118], or employing microfluidic channels within the antenna for enhanced interaction with the target analyte [23], hold promise.

Interference from other radiation sources or neighboring antennas can degrade the performance of passive antenna sensing systems. The backscattered or reflected signals from the antenna can be complex, influenced by various factors beyond the target parameter. Advanced signal processing techniques, such as machine learning and deep learning algorithms, need to be developed to effectively extract the desired information and achieve robust data interpretation [119]. Also, developing robust techniques for signal detection is essential to ensure reliable operation in complex electromagnetic ambient backscattering environments [120]. Moreover, coexistence with other wireless systems poses additional challenges that need to be addressed.

Another challenge is the cost of antenna-based sensors. Incorporating smart materials often increases the overall cost of the sensor. Manufacturing antenna-based sensors can also be expensive due to the complicated design structures, complex fabrication processes, and required substrates. Hence, choosing a suitable material is crucial for cost efficiency.

To address these technical challenges, future research efforts should focus on developing innovative antenna designs tailored to the specific requirements of passive sensing applications. This includes exploring novel materials [121], geometries, and fabrication techniques to enhance the sensitivity and resolution. Furthermore, investigating advanced signal processing algorithms is crucial for improving the detection and classification capabilities of antenna-based passive sensing systems. Machine learning approaches, such as neural networks and deep learning, hold promise for extracting meaningful information from complex electromagnetic signatures [122]. Additionally, novel fabrication techniques such as additive manufacturing and 3D printing can help reduce antenna-based sensor’s cost and manufacturing complexity [4].

Current antenna designs primarily focus on sensing a single parameter, such as temperature, pressure, or strain. Discerning between multiple parameters using a single antenna design remains a challenge. Future research should explore multi-resonant antenna structures [74] or integration with metamaterials to achieve multi-parameter sensing and improve data richness [123]. Integrating antenna sensors with other sensing modalities [124] could open doors to comprehensive sensing solutions for complex industrial scenarios. Research on co-designing and integrating these different sensing modalities is a promising future direction.

It is essential to note that the choice between a traditional and an antenna-based sensor should be based on the specific applications and requirements. In some scenarios, traditional sensors might be the preferred choice due to their simplicity, directness of measurement, or established reliability. However, antenna-based sensors are becoming increasingly attractive due to their unique features and technological advancements. By addressing the technical challenges and exploring future research directions outlined in this article are essential for unlocking its full capabilities. By fostering interdisciplinary collaborations and leveraging cutting-edge technologies, the field of antenna-based passive sensing is poised for continued advancement and innovation. The rise of IoTs and the need for more innovative sensing solutions will undoubtedly drive further advancements in this field.

## 7. Conclusions

The use of antenna-based sensors in emerging sensing devices is on the rise. It is mainly due to their lightweight nature, cost-effectiveness, and conformal characteristics, which render them ideal for wireless passive sensing needs. Starting with an overview of the antenna’s fundamental operating principle, this paper elaborates on the distinct traits that define antenna-based sensing. The influence of a material’s various attributes, including dielectric properties, conductivity, and loss tangent, on the operational effectiveness of antenna-based sensors is expounded upon. Subsequently, it examines the types of sensing techniques categorized for an antenna-based sensor and discusses the existing literature. For each sensing technique, diverse sets of reference cases are provided, contributing to a thorough grasp of the concrete utilization of these techniques. This paper concludes by addressing technical barriers, proposing potential solutions, outlining future directions, and presenting suggestions for researchers and antenna designers pertaining to the selection of material types during the manufacturing of antenna-based sensors for better performance. In conclusion, while traditional and antenna-based sensors represent significant advancements in sensing technology, they cater to different needs and applications. The choice between them would be dictated by the specific requirements of the deployment scenario and the inherent advantages and challenges associated with each technology.

## Figures and Tables

**Table 1:** Permittivity (ϵ), loss tangent (δ) values of common substrate materials for antenna fabrication [68].

Substrate Material	Dielectric Constant	Loss Tangent (*δ*)	Supplier
RT/duroid 5880	2.2	0.0009	Rogers
TLX-9	2.5	0.0019	Taconic
AD 255C	2.55	0.0014	Arlon
GML 1000	3.2	0.004	GIL
RO4003	3.38	0.0029	Rogers
TLF-34	3.4	0.0020	Taconic
AD 350A	3.50	0.0030	Arlon
FR402	4.25	0.016	Isola

**Table 3:** A comparison table of microstrip-based resonator sensors.

References	Resonator Sensor	Frequency (GHz)	Normalized Sensitivity (%)	Measurand
[111]	SRR	1.72	0.78	Microfluidic sensing
[112]	SRR	2.1	0.091	Dielectric characterization
[113]	SIW	0.0014	0.15	chemical sensing
[110]	CSRR	0.33	0.504	Liquid sensing
[114]	SRR	3	0.026	Liquid sensing
[109]	SIW	4.4	0.044	Biochemical sensing
[102]	CSRR	2.4	0.31	Dielectric characterization
